# Microbial-Mediated Plant Growth Promotion and Pest Suppression Varies Under Climate Change

**DOI:** 10.3389/fpls.2020.573578

**Published:** 2020-09-10

**Authors:** Sharon E. Zytynska, Moritz Eicher, Michael Rothballer, Wolfgang W. Weisser

**Affiliations:** ^1^ Terrestrial Ecology Research Group, Department of Ecology and Ecosystem Management, School of Life Sciences Weihenstephan, Technical University of Munich, Freising, Germany; ^2^ Department of Evolution, Ecology and Behaviour, Institute of Infection, Veterinary and Ecological Sciences, University of Liverpool, Liverpool, United Kingdom; ^3^ Institute of Network Biology, Helmholtz Zentrum München GmbH, Neuherberg, Germany

**Keywords:** aphid, barley, carbon dioxide, earthworms, ozone, PGPR, plant-insect-microbe, rhizobacteria

## Abstract

Climate change is altering the dynamics of crop pests and diseases resulting in reduced crop yields. Using beneficial soil bacterial to increase crop health is a quickly developing area in sustainable agriculture, but it is unknown if climate change or interactions with other species could alter their effect. The plant growth-promoting rhizobacterium *Acidovorax radicis* N35 is known to increase barley (*Hordeum vulgare*) plant growth under laboratory conditions, and we tested the stability of the plant-bacterial interactions when exposed to elevated carbon dioxide (CO_2_) and ozone (O_3_) levels while infesting the aboveground leaves with cereal aphids (*Sitobion avenae*) and the soil with beneficial earthworms. *Acidovorax radicis* N35 increased plant growth and reduced insect growth – with greatest effect in a high-stress elevated O_3_ environment, but reduced effects under elevated CO_2_. Earthworms promoted both plant and insect growth, but inoculation with *A. radicis* N35 alleviated some of the earthworm-mediated increase in pest abundance, particularly in the ambient environment. The consistency of these beneficial effects highlights the potential of exploiting local species interactions for predicting and mitigating climate change effects in managed systems. We conclude that microbial bioprotectants have high potential for benefiting agriculture *via* plant-growth promotion and pest suppression.

## Introduction

Climate change is predicted to expand insect pest range distributions and shift insect phenology (Tylianakis et al., 2008), resulting in increased chances of pest outbreaks. Combined with our reduced ability to control insect pests as a consequence of increasing rates of insecticide resistance (Malloch et al., 2016) and the declining biodiversity of natural enemies (Oliver et al., 2015; Seibold et al., 2019), predicted losses to crop yields are high. Further exacerbating the situation, increases in global carbon dioxide (CO_2_) and ground-level ozone (O_3_) are major concerns for crop-insect interactions (IPCC, 2019). While higher CO_2_ generally increases absolute plant growth, it also reduces plant nutrition, alters plant physiology (Fuhrer, 2003; Sun et al., 2016), and increases the growth rate of sap-feeding insects such as aphids (Robinson et al., 2012). Ground-level ozone is a known stressor that reduces plant growth and increases plant susceptibility to pests and disease (Fuhrer, 2003; Plessl et al., 2005). However, ozone can also induce plant defence pathways, e.g., PR-proteins β-1,3-glucanases and chitinases (Plessl et al., 2005), that are involved in plant resistance to sap-feeding insects (Forslund et al., 2000). Understanding the complexities of these interactions is necessary in order to predict future outcomes and develop solutions to mitigate these effects. This means going beyond studying pairwise, or even tri-trophic systems, and performing larger multi-factorial experiments that enable us to disentangle the complex interactions and identify emergent properties novel to these multi-species communities (Levine et al., 2017). A biodiverse and well-functioning ecosystem consists of many different species of microorganisms, plants, and animals, each performing specific functions (Meyer et al., 2018). Management of cropping systems disrupts these natural processes (Seibold et al., 2019), but one solution is to identify and promote beneficial interactions that can buffer the effects of climate change on crop plants. There is already much research on adapting agricultural landscapes to promote the services of pollinating insects (Pufal et al., 2017), and this can have a knock-on effect for natural enemies of pests (Balzan, 2017). An extensive range of bioprotectant technologies are being developed, and the emphasis is on considering plant protection in a multitrophic, whole ecosystem context (IBMA, 2018). While bioprotectants can be microorganisms, semiochemicals, plant extracts, or natural substances, the main focus is on providing holistic solutions with negligible harm to the environment (Bender et al., 2016; Backer et al., 2018; IBMA, 2018). The use of bioprotectants is very promising, and we expand upon this to ask if these beneficial effects are maintained across different biotic and climate environments.

Soil microbes have already been shown to affect how the plant responds to aboveground pest insects (Pieterse et al., 2014; Pineda et al., 2017). For example, inoculation of *Arabidopsis* roots with *Bacillus velezensis* reduced the feeding and growth rates of *Myzus persicae* aphids (Rashid et al., 2017) and arbuscular mycorrhizal fungi (*Rhizophagus irregularis*) induced resistance in potato plants to the cabbage looper (*Trichoplusia ni*) (Schoenherr et al., 2019). Promoting interactions that both directly increase plant growth and also help reduce pest populations would provide a win-win solution for agriculture (Pineda et al., 2017). Understanding if soil microbes provide a solution for agriculture under global change requires exposing them to plants under climate change conditions and challenging the plant microbe interactions with other organisms including pest insects, but also other soil biota (Levine et al., 2017). While elevated CO_2_ and O_3_ generally do not have strong direct impacts on soil microbial communities, the abundance of nitrogen fixing bacteria may increase as a response to increased plant productivity under elevated CO_2_ (Wang et al., 2017) and decrease as a response to reduced plant growth under elevated O_3_ (Changey et al., 2018). Such interactions can further enhance or disrupt the effect of any inoculated bacteria. Similarly, the interaction with other soil biota is also important, as microbe-plant interactions may be very different in sterilized vs. live soil. In addition, a number of other soil organisms such as earthworms are known to also affect plant-insect interactions and potentially enhance or decrease any effect of particular microbes on the plant (Braga et al., 2016; Xiao et al., 2018).

We used a cereal barley crop system where we inoculated four cultivars (*Hordeum vulgare* L.; cv Barke, cv Chevallier, cv Grace, cv Scarlett) with the rhizobacterium *Acidovorax radicis* N35 (herewith, *A. radicis*) that was first isolated from cereal plants and has shown to promote shoot and root growth (Li et al., 2012; Han et al., 2016). The barley cultivars were chosen as they varied in their effect on aphid (*Sitobion avenae*) growth rate in preliminary experiments. We used unsterilized potting soil to study the effect of *A. radicis* inoculation on changes in the soil community, as well as on plant growth and aphid pest suppression. We also added earthworms (*Dendrobaena veneta*) that alter plant-aphid interactions (Singh et al., 2014) and can mediate interactions with the root-associated microbiota (Braga et al., 2016). Under a fully-factorial experimental design, the plants were grown in four climate environments (ambient, elevated CO_2_, elevated O_3_, and combined eCO_2_+eO_3_), across three separate (temporal) runs allowing for full replication across four climate chambers (**Figure S1**).

We asked if the strength of the effect of the inoculated rhizobacteria on plant growth would change across the various treatments, in particular whether it holds both in the higher-stress environments (i.e., elevated ozone and aphid infestation) and the lower-stress environments (i.e., control, elevated CO_2_ or earthworm environments). We also tested if the interaction with earthworms increases or decreases any microbe effect on plants, and if the effect was consistent across different plant cultivars.

## Materials and Methods

### Study System

Our study species included: (1) four European barley (*Hordeum vulgare*) plant cultivars: Barke (Saatzucht Breun GmbH), Chevallier (New Heritage Barley Ltd), Grace (Ackermann Saatzucht GmbH), and Scarlett (Saatzucht Breun GmbH); (2) the English grain aphid *Sitobion avenae* (L.) that had been maintained as low density stock populations on Barley cultivar “Kym” in a climate cabinet for two years, the clone was originally from Goettingen University; (3) epigeic earthworms *Dendrobaena veneta* Rosa 1886, originally from wurmwelten.de and maintained in a Worm-Café^®^ for three years prior to the experiment; and (4) the rhizobacteria *Acidovorax radicis* N35 prepared by colleagues from the Helmholtz Zentrum Munich, along with a control solution containing no bacteria for seedling inoculation.

### Experimental Design

The climate experimental treatments [carbon dioxide, CO_2_ (elevated/ambient), ozone, O_3_ (elevated/ambient)] were used at the level of an individual climate chamber with four chambers used: (1) ambient (~500 ppm day-time during high light periods, 600 ppm night-time; 0.02 ppb ozone), (2) elevated CO_2_ (700 ppm day-time during high light periods, 900 ppm night-time), (3) elevated O_3_ (constant 100 ppb), and (4) elevated CO_2_ and elevated O_3_. The experiment was run across three successive temporal blocks (runs), and chamber identity was changed across runs, such that each climate treatment was run in three different chambers across the experiment to avoid a chamber-treatment confounding effect.

The biotic experimental treatments [plant cultivar (Barke, Chevallier, Grace, Scarlett), *A. radicis* (presence/absence), earthworms (presence/absence), aphids (presence/absence)] were run at the level of an individual pot within a chamber. Within each run, three replicates of each biotic (plant cultivar, *A. radicis*, earthworm, aphid) treatment were made with each replicate allocated to one of three tables (randomized block design within run, within chamber). The total number of replicates in the design was nine, three per treatment per run.

The experimental design was fully-factorial, with three temporal blocks (runs) and blocks within chambers (tables). Table within chamber was not a significant block effect, indicating the high homogeneity of the climate chambers.

### Experimental Set-Up

Seeds were germinated between moistened filter paper for 5 days in the dark at room temperature. After this the seedlings were soaked in either *A. radicis*-containing solution or control solution for 1 h. *A. radicis* was grown by inoculating the surface of NB plates, and incubated at 30°C for 36 h. Then the cultures were resuspended in 10 mM MgCl_2_ with final suspension containing 10^9^ cells per ml. The control solution was 10 mM MgCl_2_, and 100 µl Tween 20 was added to both bottles. Before transplantation, the length of the shoot and longest root of the seedlings was measured. Then, seedlings were planted into 10 cm pots (single seedling per pot) containing soil substrate (Floragard B Pot Medium-Coarse, pH 5.6, NPK 1-0.6-1.2) mixed with quartz sand at a 5:1 (soil:sand) ratio. Plants grew uncovered for three days, when shoot length (from top of the seed to the longest leaf) was again measured. Aphids were introduced to plants using a fine paintbrush to move two 4^th^ instar aphids from the stock populations (kept at low densities to avoid winged aphid production) onto the base of the plant shoot. From here, aphids will move up onto the plant where they feed, develop into adults, and then begin to produce offspring within the next few days. Earthworms were first washed in tap water and placed into plastic tubs with moist tissue for 48 h to remove gut contents. Then, five worms were introduced into the soils (at the same time as aphid infestation), with a total biomass 1.1–2.1 g (biomass recorded).

All pots were covered with a 180 x 300 mm air-permeable cellophane cover (HJ Kopp GmbH, Germany) on the top, and organza mesh at the base of the pot, secured by two elastic bands. Plants were allowed to grow for 14 days under 20°C, 65% RH (relative humidity), with 10 h of full light (850 PAR), 8 h of total darkness and a 3-h sunrise/sunset gradient between these where light was gradually increased/decreased. At the end of the experiment, aphids were counted using hand tally-counters, ensuring a systematic method of counting each leaf from the base to the top. Plant shoot length (longest leaf) and root length (longest root) were measured; barley plant shoot and root length during the experimental period is a good predictor of dry biomass and final yield (**Figure S2**). Earthworms extracted from the soil were washed, counted, and earthworm biomass measured. All five earthworms were recovered from 95.6% of pots, with only 2.5% of pots containing fewer than four earthworms (13/522 pots). Root material was collected and stored at -20°C before DNA extraction for microbial community analysis.

### Phenotypic Data Analysis

Two approaches were used to analyse the phenotypic experimental data. All data were analysed in R 3.5.1 using RStudio (Version 1.1.463). The first approach used standard linear models for variance partitioning of the data (N=986; 4–11 replicates per treatment; **Figure S1**), where model response variables were (1) seedling viability: longest shoot length at day 8 minus longest shoot length at day 5 (cm), (2) plant growth: longest shoot length at day 22 minus longest shoot length at day 8 (cm), (3) Root growth: longest root length at day 22 minus longest root length at day 5, (4) aphid density: total number of aphids divided by the plant growth variable (day 22–day 8) giving the number of aphids per cm of plant. All models included the experimental run as a blocking factor to control for variation across the three temporal blocks. Diagnostic plots of the models showed that standard linear models with a normal error distribution were suitable for all variables. Initial models included all main effects and interactions, and were simplified using a backwards stepwise method removing the least significant interaction terms one by one until a minimal adequate model is reached.

The second method focused on the effect of *A. radicis* inoculation on the same variables as above. However, here we used a matched pairs analysis that matched plants within treatments that had been inoculated with *A. radicis* compared to controls (N=474 pairs). We took care to only match plants from the same tables (achievable due to the rando.mized complete block experimental design used) to minimize differences due to variation within a chamber or across temporal runs. The absolute differences between these plants for each of the variables (seedling viability, plant growth, root growth, and aphid number) were then used to calculate the log-response ratio (lnRR, treated vs control). The lnRR values were then analyzed using linear models using all main effects and interactions, thus determining the impact of these on the effect size (strength and direction) of *A. radicis* inoculation. Figures use the calculated mean effect size (lnRR) across treatment combinations and the associated variance (using the R package “metaphor”).

### Microbial Community Barcoding

To assess the root-associated microbial community, 0.25–0.5 g of roots with attached soil was used for DNA extraction (Qiagen DNeasy PowerSoil Kit). The DNA extraction, amplification and sequencing were performed by AIM (Advanced Identification Methods GmbH, Munich). The V3-V4 region of the 16S rRNA gene was amplified using primers 341f (CCTACGGGNGGCWGCAG) and 785r (GACTACHVGGGTATCTAATCC), which showed the best coverage for bacteria and was most reproducible in a recent comparative evaluation (Thijs et al., 2017). A total of 7,382,326 paired end reads were recovered, with a median of 92.8% reads merged. Sequence data processing was performed using the IMNGS platform(Lagkouvardos et al., 2016) applying the UPARSE amplicon analysis pipeline(Edgar, 2013). Statistical evaluation was done with the Rhea pipeline for R (Lagkouvardos et al., 2017). The datasets supporting the conclusion of this article are available through GenBank.

## Results

**Figure 1:**
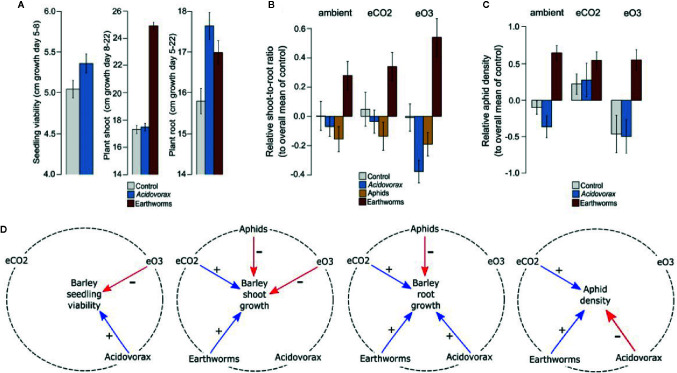
Plant and insect growth across biotic and climate treatments. **(A)** Absolute early seedling growth (seedling shoot length difference, day 5–8, cm), later shoot growth (longest shoot length difference, day 8–22, cm), and root length (day 5–22, cm) across *Acidovorax radicis* and earthworm treatments, averaged across all barley cultivars. **(B)** Relative shoot-to-root ratio and **(C)** relative aphid load (compared to controls, by plant cultivars and experimental runs), across abiotic (climate) and biotic (*A. radicis*, aphids and earthworm) treatments. Error bars are ±1 SE **(D)** Summary of interactions showing positive effects (+) in blue (on the variable in the centre of each circle), negative effects (-) in red, and the dotted line shows the factors linked by interactions.

We found overall positive effects of the inoculated rhizobacterium *Acidovorax radicis* N35 on plant root and shoot length (growth promotion; **Figure 1A**) altering the allocation of energy between plant shoot and roots (shoot-to-root ratio; **Figure 1B**), and negative effects on aphid density (aphid suppression; **Figure 1C**). While our analyses uncovered multiple interactions between the climate and biotic factors on plant growth and aphid density (**Figures 1B, C**; **Table S1**), meaning that the effect of one factor depended on others, most of the results could be simplified to a set of factors that together are important for the outcome (**Figure 1D**).

The presence of the inoculated rhizobacterium *A. radicis*, elevated CO_2,_ and earthworms increased plant growth, whereas elevated O_3_ and aphids decreased plant growth (**Figure 1D**; **Table S1**; **Figure S3**). *Acidovorax radicis* had a stronger growth promotion effect on the plant roots than on aboveground tissues (**Figure 1A**) leading to reduced shoot-to-root ratio on inoculated plants (**Figure 1B**). The presence of aphids aboveground decreased shoot growth leading to a reduction in plant shoot-to-root ratio, while belowground earthworms promoted shoot growth (**Figure 1A**) driving a strong increase in shoot-to-root ratio (**Figure 1B**). While *A. radicis* reduced aphid density in general, elevated CO_2_ and earthworms increased aphid density on the plants across all cultivars (**Figure 1D**; **Table S1**; **Figure S3D, G**).

**Figure 2:**
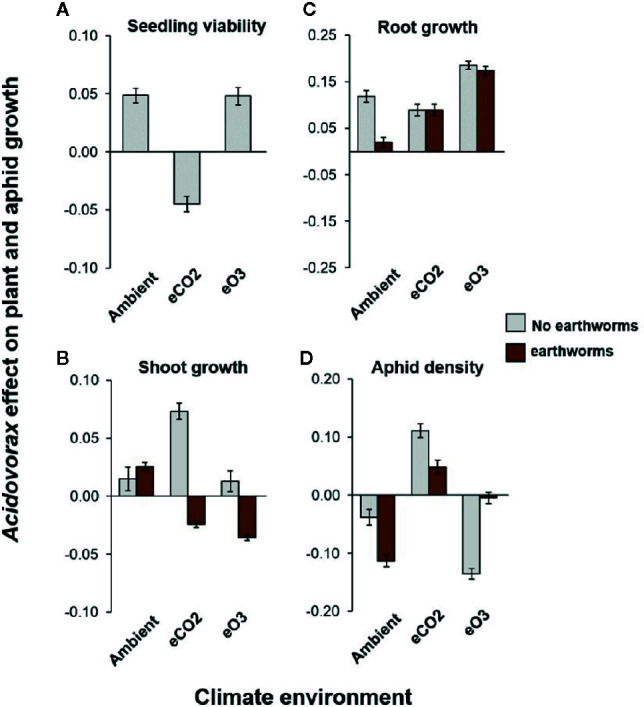
Effect of *A. radicis* inoculation from paired analysis. Data shows the log-response of plant and aphid growth traits comparing plants that were treated with *A. radicis* with one treated with a control solution, for **(A)** seedling viability (early shoot growth), **(B)** shoot growth, **(C)** root growth, and **(D)** aphid density. Error bars show the variance around this ratio. N = 474 pairs.

We used matched pairs analysis to analyse how the benefits of *A. radicis* inoculation varied across the climate and biotic environments, by comparing responses of control to treated plants (**Figure 2**; **Table S2**; **Figure S4**). Both under an ambient and stressed elevated O_3_ environment, *A. radicis* was overwhelmingly beneficial for the plant by increasing seedling (**Figure 2A**) and root growth (**Figure 2C**) while reducing aphid numbers (**Figure 2D**). However, under elevated CO_2_ (which benefitted both plant and aphid growth), the beneficial effect of *A. radicis* on seedling growth (**Figure 2A**) and pest suppression (**Figure 2D**) was no longer visible, yet there was a positive effect on later shoot growth (**Figure 2B**). Thus, the timepoint at which *A. radicis* influences plant growth is dependent on the environment, with knock-on effects for pest suppression effects.

The presence of earthworms and *A. radicis* individually promoted total plant growth (**Figure S5**), with earthworms increasing shoot growth more than root growth and *A. radicis* promoting root growth (**Figures 1A, B**). The effect of earthworms and *A. radicis* was not strictly additive but the relationship was also not overwhelmingly interactive resulting in weak higher-order interactions explaining the effect of *A. radicis* on plant shoot and root growth (**Table S2**). In general, the effect of *A. radicis* only depended on the presence of earthworms in specific examples, e.g., root growth in ambient environment, and shoot growth in elevated CO_2_ or elevated O_3_ (**Figures 2C, D**).

In the ambient and elevated O_3_ environments, *A. radicis* inoculation reduced aphid density with up to 10% pest suppression effect (**Figure 2D**). However, under elevated CO_2_ this pest suppression effect was lost. Earthworms increased the pest suppression effect of *A. radicis* by further decreasing aphid density in an ambient environment but not under elevated O_3_ where the effect was stronger without them (**Figure 2D**; **Table S2**). The loss of pest suppression by *A. radicis* under elevated CO_2_ was mitigated by the presence of earthworms that reduced the positive effect seen of inoculation in this environment (**Figure 2D**; **Table S2**). Thus, the pest suppression effect was stronger when earthworms were present (except under elevated O_3_), and earthworm presence would be expected under field conditions.

We observed variation in the response to *A. radicis* across plant cultivars (**Figure S4**). While the overall response to *A. radicis* was positive for total plant growth (**Figure S5**), some cultivars experienced the greatest benefit during seedling growth (cv *Barke* and cv *Scarlett*) whereas cv *Chevallier* responded primarily through increased root growth (**Figure S4**). cv *Grace* showed highest beneficial effects of *A. radicis* under the eO_3_ stress environment and a negative response under elevated CO_2_ (**Figure S4**). Similarly, the response of aphids to *A. radicis* also varied across the cultivars. The average effect was for pest suppression in the ambient environment, yet cv *Barke* and cv *Grace* showed opposite patterns in their response to earthworms with greater pest suppression with earthworms on cv *Barke* while this happened in the absence of earthworms for aphids on cv *Grace* (**Figure S4**). The loss of pest suppression under elevated CO_2_ was primarily driven by aphids responding positively to *A. radicis* on cv *Grace* while aphids on the other cultivars (cv *Barke*, cv *Scarlett*) had a reduced response to *A. radicis* in this environment (**Figure S4**).

**Figure 3:**
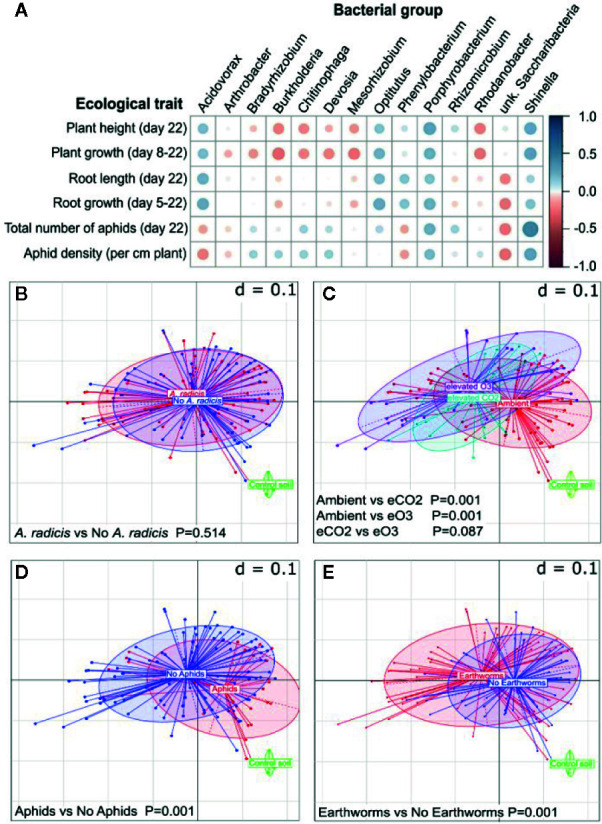
Changes in the plant root microbiome (16S analysis). **(A)** Correlation plot showing negative (red) and positive (blue) correlations between different measured plant parameters and the abundance of detected genera. The bigger the circle the higher the significance (i.e., the lower the p-value), cutoff was set to p=0.05. **(B**–**E)** Multi-dimensional scaling plots of microbial profiles with d=0.1 meaning that the distance between two grid lines represents approximately 10% dissimilarity between the samples, when separated by **(A)** *A. radicis* inoculation, **(B)** climate treatment, **(C)** aphid infestation, and **(D)** earthworm addition.

The microbial community analysis confirmed the presence of *A. radicis* in the rhizosphere at the end of the experiment and showed that increased abundance of *A. radicis* was correlated with increased plant growth and decreased aphid densities (**Figure 3A**). Overall, the inoculation of *A. radicis* did not significantly alter the bacterial community on the barley roots (**Figure 3B**). In contrast, the climate environment (**Figure 3C**), aboveground aphid feeding (**Figure 3D**), and the presence of earthworms in the soil (**Figure 3E**) significantly changed the root-associated microbial community. Thus, *A. radicis* did not dominate the root microbiome, and does not need to since it had strong ecological effects at low abundance. Such traits are desirable for plant-growth-promoting rhizobacteria. This data also shows that other biotic and abiotic factors have a much stronger effect on the plant microbiome, potentially allowing the plant to adapt to adverse conditions through recruitment of other beneficial bacteria.

The abundance of *Phenylobacterium* was also correlated with higher plant growth and lower aphid densities (**Figure 3A**). Some groups including *Shinella* and *Porphyrobacter* bacteria were correlated with increased growth of both the plant and the aphid, and an unknown Saccharibacteria was correlated with reduced plant and aphid growth. Several bacterial groups, including *Burkholderia*, were negatively correlated with plant growth, but somewhat positively with aphid abundance. Such effects are undesirable in crop microbiomes, and subsequent analysis showed that *A. radicis* inoculation and earthworms were correlated with a reduced abundance of *Burkholderia* (**Figure S6**). However, the main effects of *A. radicis* on the plant and pests is likely *via* a direct interaction with the plant rather than mediated by changing the soil microbial community.

## Discussion

Our results show that the effect of *A. radicis* N35 inoculation was overall positive for plant growth and pest suppression (negative effect on aphid density). The strength, and in specific examples the direction, of these effects varied with the biotic (cultivar and earthworm) and climate (elevated CO_2_ and O_3_) environment. *Acidovorax radicis* was more beneficial to the plant under a stressed environment, with increased positive effects on root growth under elevated O_3_ and stronger pest suppression in the absence of earthworms. Surprisingly, the addition of *A. radicis* to the plants did not alter their overall root-associated microbial community, which means this bacterium has strong ecological effects without dominating the native community. Yet, infestation by aphids and earthworms, and the climate environment significantly changed the microbial community.

The growth promotion effects of *A. radicis* on the plant root and shoot were not unexpected (Li et al., 2012; Han et al., 2016), and we found that the strongest effects occur in the very early stages of plant growth and on root growth in the ambient and elevated O_3_ environments. Under elevated CO_2_ *A. radicis* had a reduced effect on the early growth of the plant but a stronger positive effect on later growth. It is possible that the increased CO_2_ enabled the plant to benefit from the bacterium in ways that were not possible in the other environments. For example, a study on phytoremediation found that the beneficial effects of a rhizobacteria were enhanced under elevated CO_2_ and was attributed to the regulation of photosynthesis (Guo et al., 2014). While plant vegetation growth does not necessarily equate to increased plant fitness, we have shown in additional experiments that this increased early growth is positively correlated with seed mass (**Figure S2D**). Our experiment analysed plant growth up to day 21, which is during tillering and before stem elongation. Next important steps in this research include determining at which stage the plant responds most strongly to *A. radicis* inoculation, or if the response of the plant varies across growth stages. This is of particular interest when considering future applications of microbes to crop plants, for example when a single inoculation during germination provides all the benefits required by the plant with no need for a second application (Backer et al., 2018). While earthworms themselves strongly increased plant growth, our results suggest that they do not interfere with the beneficial effects of *A. radicis* (weak higher-order interactions). Previous work has found synergistic effects of rhizobacteria and earthworms, suggesting that in a wider community adaptation may occur over longer time periods (Braga et al., 2016). The substantial variation across the four barley cultivars in their responses to the different environments (**Table S1**) was predominantly through differences in the strength of effects; yet, instances certainly occurred where the barley cultivars responded in contrasting ways. While variation among cultivars can reduce the predictability of effects across a wider range of barley cultivars, we can harness this variation for future comparative analyses. By using contrasting cultivars, we can better understand the molecular mechanisms underlying these differences in future work; a similar approach as when comparing wild-type strain to mutants, or old landrace cultivars to modern ones.

Our measure of aphid density (number of aphids per cm of plant) controlled for the increased plant growth due to elevated CO_2_ and earthworms; thus, any changes in aphid density occurred through plant physiological changes such as the abundance of amino-acids or defence signalling (Ryan et al., 2015; Sun et al., 2016; Mur et al., 2017; Xiao et al., 2018). The reduction in aphid density on *A. radicis* inoculated plants is consistent with other studies finding similar pest suppression effects (Gadhave et al., 2016; Rashid et al., 2017; Pineda et al., 2017). Expected mechanisms for these effects include induced systemic resistance (ISR) in the plant (reviewed by Pieterse et al., 2014) where the inoculated bacteria alter plant signalling hormones (e.g., JA, SA, SBA) leading to higher resistance against the feeding aphids. Alternatively, other molecular pathways in the plant could be switched on resulting in reduced aphid feeding or aphid growth. For example, Rashid et al. (2017) found that inoculation of *Arabidopsis* with *Bacillus velezensis* did not alter any ISR-related responses but increased callose deposition onto phloem sieve tubes, which inhibited the aphids from ingesting the phloem sap. Ongoing work will identify the molecular mechanisms involved, but we highlight the importance of following whole-genome approaches on which to identify novel pathways of importance rather than focusing only on “popular” mechanisms. Our study is the first to show that the pest suppression effect varies across climate environments, plant cultivars, and due to the presence of earthworms in the soil. Elevated CO_2_ provided the only environment where the general effect was for an increase in aphid density after inoculation with *A. radicis*, but this increase was mitigated by the presence of earthworms. In the ambient environment, earthworms even increased the reduction of aphids by *A. radicis*. Earthworms are ecosystem engineers and have been shown before to alter plant-aphid interactions (Singh et al., 2014); however, there are numerous ways in which this can happen. Earthworms may alter the environment around the root (nutrients, oxygenation) or alter plant defence chemicals, and have been posited as potential vectors of PGPR (positive) or selective filters for inoculated bacteria (negative) in agricultural systems (Suarez et al., 2014; Xiao et al., 2018).

We showed that the microbial community of the plant roots was not altered due to inoculation of *A. radicis*. The roots were only sampled at the end of the experiment, providing a single time point for the bacterial community analysis. For a more in-depth understanding of the microbial community dynamics we would need to run a time-series analysis, which would further provide information on how the abundance of *A. radicis* changes after inoculation. As bioprotectants, beneficial bacteria would be inoculated into field soils that already have a native microbiome and using bacteria that do not disrupt the native community are advantageous while maintaining their benefits. Additionally, a combination of beneficial bacterial strains might be used to obtain multiple benefits in maximizing plant defences and yield. However, previous work has found mixed results when using multiple bacteria, often with no stronger effect in plant growth or yield than when one of the strains is inoculated individually. For example, the inoculation of four bacteria (*Bacillus pumilus*; *Bacillus amyloliquefaciens*; *Bacillus mojavensis*; *Pseudomonas putida*) showed minimal effects on plant growth/yield and even growth/yield reduction when double infections were inoculated (He et al., 2019). Another study on mixtures of *Bacillus* sp. also found that while individual inoculation of all strains provided positive plant effects through pest suppression, this no longer occurred when a mixture was inoculated (Gadhave et al., 2016). It is possible that these bacteria interact with the plant similarly, and the application of more divergent bacteria could help this. Alternatively, we also need to consider identifying other bacterial groups that can benefit plants but not affect or be affected by other bacteria.

A main message of our research is that while higher-order interactions can be identified using multifactorial experiments, we can also use this data to show when these are changing the direction of a response or just altering the strength of the response. This is important for understanding and predicting future outcomes across variable environments (Levine et al., 2017). We conducted this experiment across three temporal blocks which means that any significant result indicates high consistency across these replicates. The use of multiple abiotic and biotic factors, across multiple temporal blocks, will inevitably increase the size of the experiment and workload but also increases the ability to understand how this variation impacts focal interactions beyond solely predicting this from pairwise results.

In conclusion, our study showed that there is real promise for introducing beneficial soil species to benefit crop growth and simultaneously reduce insect pests in sustainable agriculture. The context-dependency of the interactions across different climate and biotic environments was found to alter the strength of effects rather than the direction. This is important since complex interactions can lead to unpredictability in outcomes, yet we found that increasing complexity (diversity) of a system had overall beneficial outcomes. Under certain environments, only one beneficial species was required yet for many others a combination of species was beneficial for plant health. While we focus on elevated ozone and carbon dioxide as abiotic factors, many other climate change related factors can have strong effects on plant-insect-microbe interactions. For example, the effect of beneficial microbes is expected to be stronger in low nutrient soils (Etesami and Adl, 2020) and under drought conditions (Rubin et al., 2017). This further suggests that beneficial microbes can have strongest effects when a plant is under stress. We highlight the need to include the effects of biotic and climate factors when developing knowledge-based ecological solutions in agriculture, and using soil organisms as bioprotectants is a promising path towards achieving low-input agriculture (Calvo et al., 2014; Bender et al., 2016; Backer et al., 2018).

## Data Availability Statement

The 16S molecular datasets presented in this study can be found in online repositories. The names of the repository/repositories and accession number(s) can be found below: https://www.ncbi.nlm.nih.gov/genbank/, MN194747 - MN195111.

## Author Contributions

SZ and WW designed the experiment. SZ and ME collected the experimental data. SZ analyzed the experimental data and MR analyzed the microbial community data. All authors interpreted the results, and SZ wrote the manuscript with all authors commenting. All authors contributed to the article and approved the submitted version.

## Funding

WW, MR, and SZ received funding from the German Research Council, Deutsche Forschungsgemeinschaft (DFG) - Project number 397565003 and SZ to the BBSRC (UKRI) *via* a David Phillips Fellowship BB/S010556/1.

## Conflict of Interest

The authors declare that the research was conducted in the absence of any commercial or financial relationships that could be construed as a potential conflict of interest.
