# Supplementary material for: Microbial-Mediated Plant Growth Promotion and Pest Suppression Varies Under Climate Change
Source: Front Plant Sci. 2020 Sep 10;11:573578. doi: 10.3389/fpls.2020.573578 (PMC7511531; doi:10.3389/fpls.2020.573578)
Supplement: Supplementary file 1 [file DataSheet_1.pdf]

SUPPLEMENTARY INFORMATION

Microbial-mediated plant growth promotion and pest suppression varies under climate change

Sharon E. Zytynska[1,2*], Moritz Eicher[1], Michael Rothballer[3], Wolfgang W Weisser[1]

[1] *Terrestrial Ecology Research Group, Department of Ecology and Ecosystem Management, School of Life Sciences Weihenstephan, Technical University of Munich, Hans-Carl-von-Carlowitz-Platz 2, 85354 Freising, Germany*

[2] *Department of Evolution, Ecology and Behaviour, Institute of Infection, Veterinary and Ecological Sciences, University of Liverpool, Liverpool, UK*

[3] *Helmholtz Zentrum München GmbH, Institute of Network Biology, Ingolstädter Landstr. 1, 85764 Neuherberg, Germany*

*Corresponding author: Sharon.zytynska@liverpool.ac.uk

Figure S1. Summary of the experimental design
Figure S2: Predictive power of plant shoot length on plant aboveground biomass and yield.
Figure S3. Main effects (averaged across barley cultivar)
Figure S4. Paired analysis of *A. radicis* (treated vs control) on plant growth and aphid density
Figure S5. Total plant growth (shoot + root growth) for each barley cultivar within each abiotic environment, across the different *Acidovorax* and earthworm treatments
Figure S6. Serial Group-Comparison depicted as box plots for *Burkholderia* abundance

Table S1. Summary of linear model results for the plant and aphid response variables
Table S2. Summary of matched pairs analysis for effect of *Acidovorax* on plant and aphid response variables

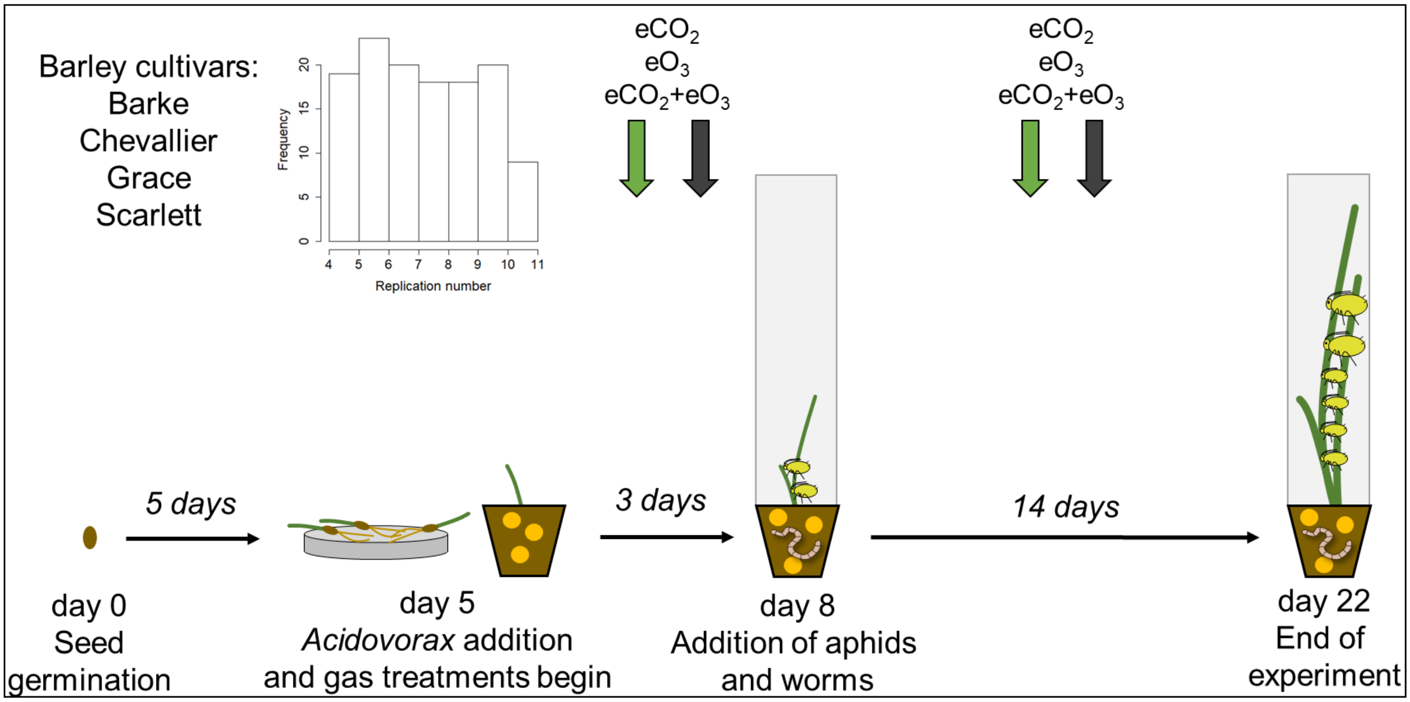

Figure S1. Summary of the experimental design. Inset shows the distribution of the number of replicates for each treatment across the experiment, with a mean of 7.7, minimum of 4 and maximum of 11. Reasons for reduced replication are from low germination of one barley cultivar, and lack of growth of the barley after transplantation, which was not linked to any experimental treatment.

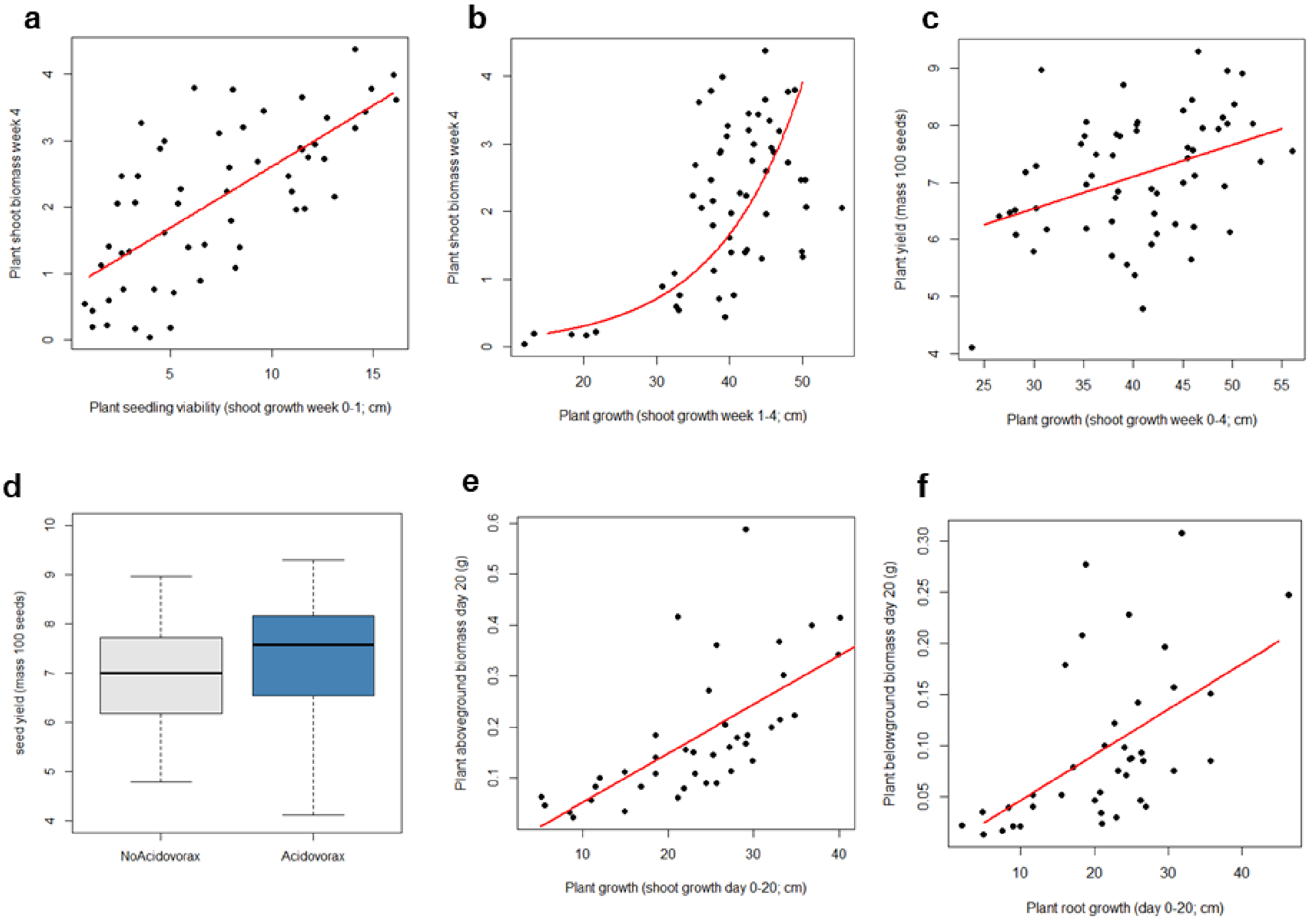

Figure S2: Predictive power of plant shoot length on plant aboveground biomass and yield. Plant growth is measured by plant height difference (longest leaf) from day 0-8, day 8-28, and day 28-56, and as dry total aboveground biomass at day 28 and 56. Plant yield is the mass of 100 seeds at day 84. In a preliminary experiment, (a) early seedling growth (day 0 to day 8) was a good predictor of plant total aboveground biomass at week 4 (day 0-8: $F_{1,55}$=52.30, P<0.001). (b) Aboveground shoot growth from day 8 to day 28 was also a good predictor of plant aboveground biomass (day 8-28: $F_{1,55}$=88.98, P<0.001), however this fitted a logarithmic relationship since plants that grew less than 30 cm in this time produced low biomass (suggesting that there is a threshold of plant height growth for biomass development). This effect changed direction after four weeks of growth (day 28-56), likely associated with changes in resource allocation as the plant begins to produce flowers and seed. (c) Early plant growth (day 0-28) was also associated with final plant yield (mass of 100 seeds) ($F_{1,58}$=10.40, P=0.002), while late plant shoot growth (day 28-56) was not associated with plant yield ($F_{1,58}$=1.14, P=0.289). (d) We also showed that inoculation of *Acidovorax* increased final plant yield (mass of 100 seeds; $F_{1,50}$=5.44, P=0.024).

A second experiment confirmed these associations. (e) Plant shoot growth from day 0 until day 20 could predict plant dry biomass at day 20 ($F_{1,38}$=31.81, P<0.001). Further, (f) root length growth and root dry biomass were also highly correlated, indicating a similar predictive power at day 20 for root length to root biomass ($F_{1,37}$=15.77, P<0.001). These results highlight the importance of early plant growth for increased yield, and due to this the current experiment focuses on the effect of the experimental treatments on early growth of the barley plants from germination (day 5-8) to day 22. Plant height difference is used to compare seedling viability (day 5-8, i.e. post-germination), and plant growth (day 8-22).

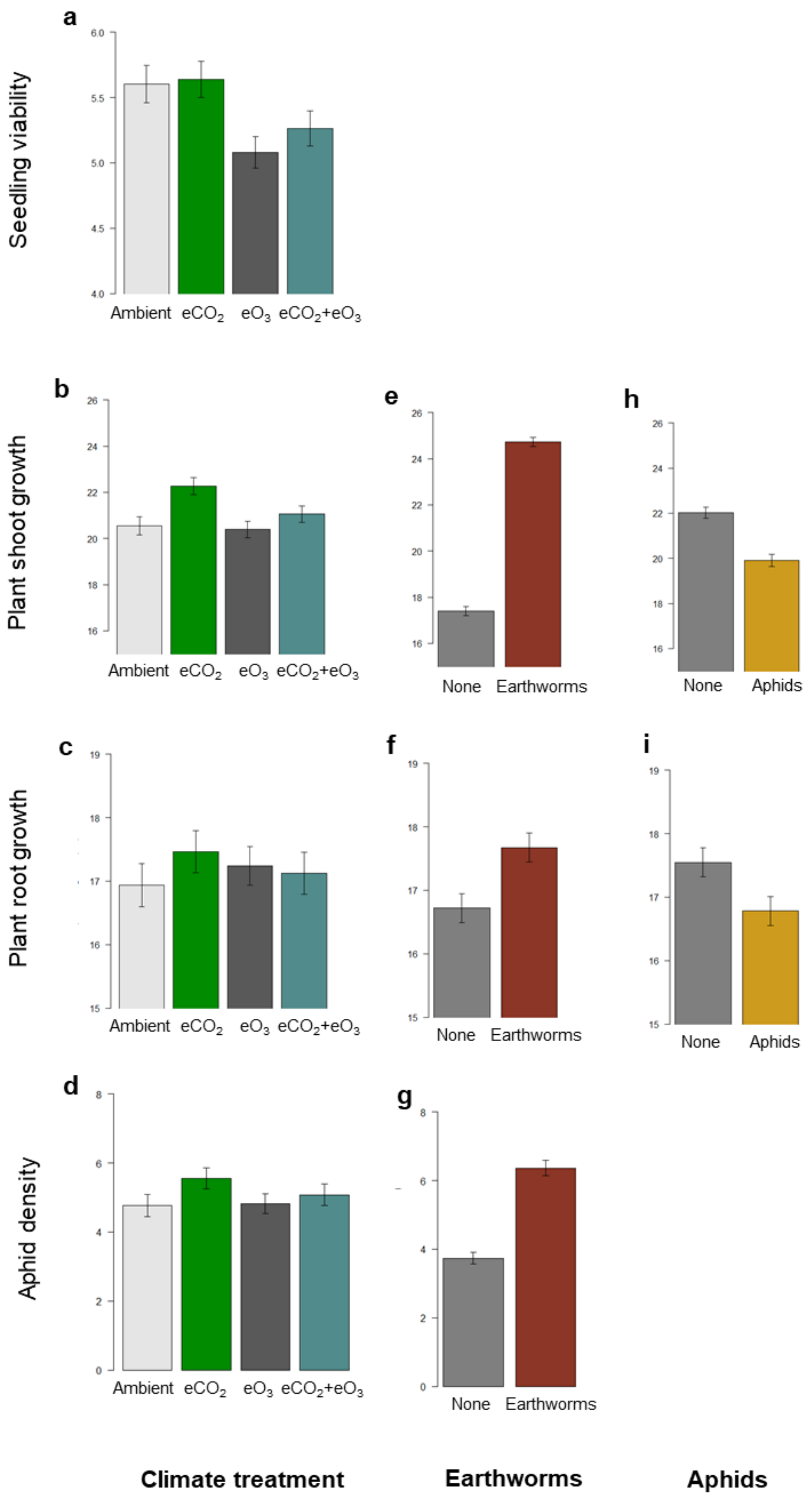

Figure S3. Main effects (averaged across barley cultivar) of abiotic treatment (ambient, elevated CO_2, elevated O_3 and combined elevated CO_2 and O_3) on plant growth (a-c) and aphid density (d), earthworms (absence/presence) on plant growth (e, f) and aphid density (g), and aphids (absence/presence) on plant growth (h, i). See Extended Data Table 1 for full results. Error bars +/- 1SE.

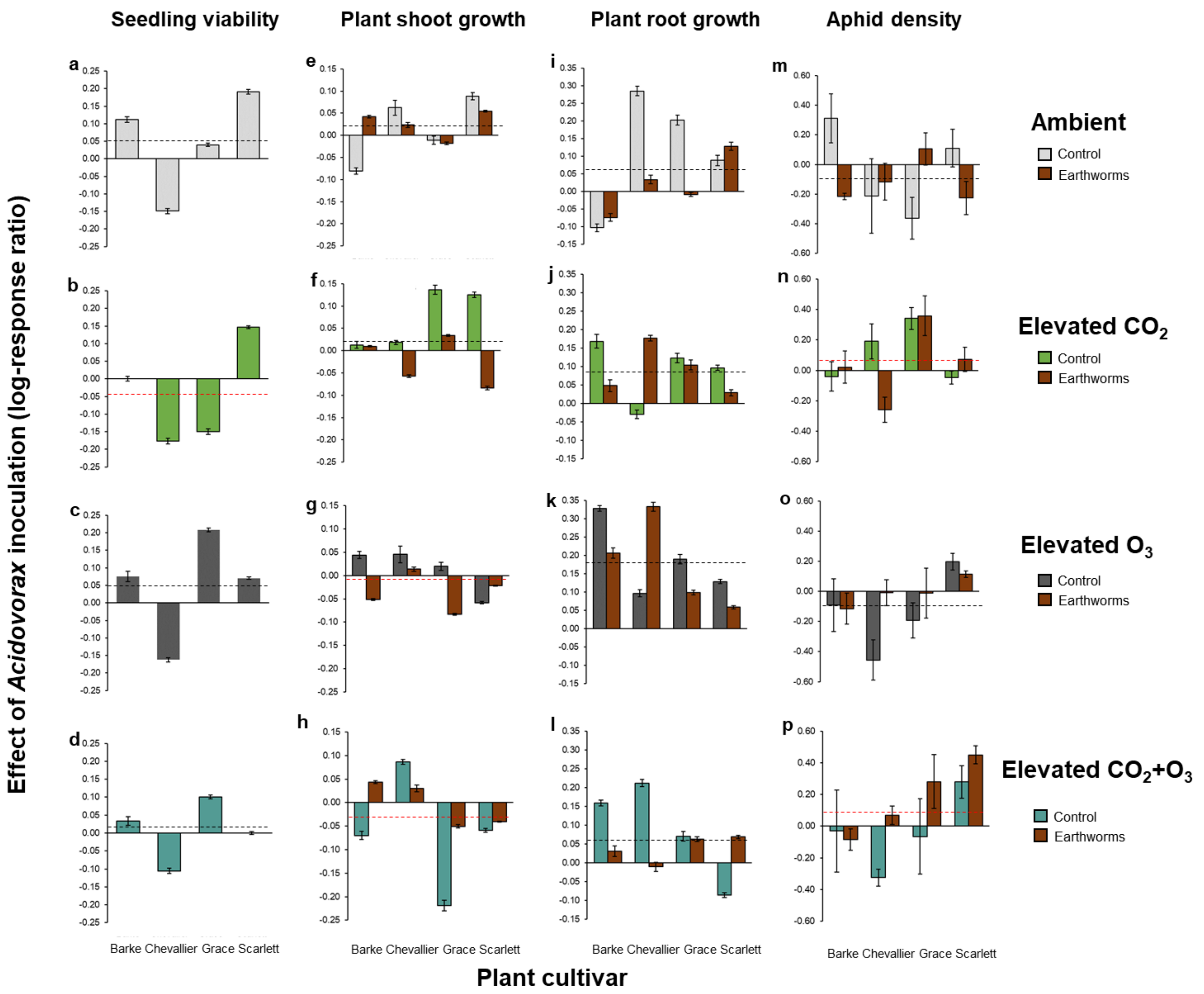

Figure S4. Paired analysis of *A. radicis* (treated vs control) on plant growth (a-l) and aphid density (m-p) across abiotic treatments. Data show effect sizes (log-response ratio) and associated variance estimates (error bars) for each barley cultivar (Barke, Chevalier, Grace, Scarlett) and earthworm treatment (clustered bars, right hand bar is earthworm presence). A positive effect size shows a beneficial effect of *A. radicis* on the measured variable, where an effect size of 0.1 represents a response of ~10%. The average effect size across all barley cultivars is shown by the dashed line (black when positive and red when negative for plant health). There was no earthworm treatment for the seedling viability measure.

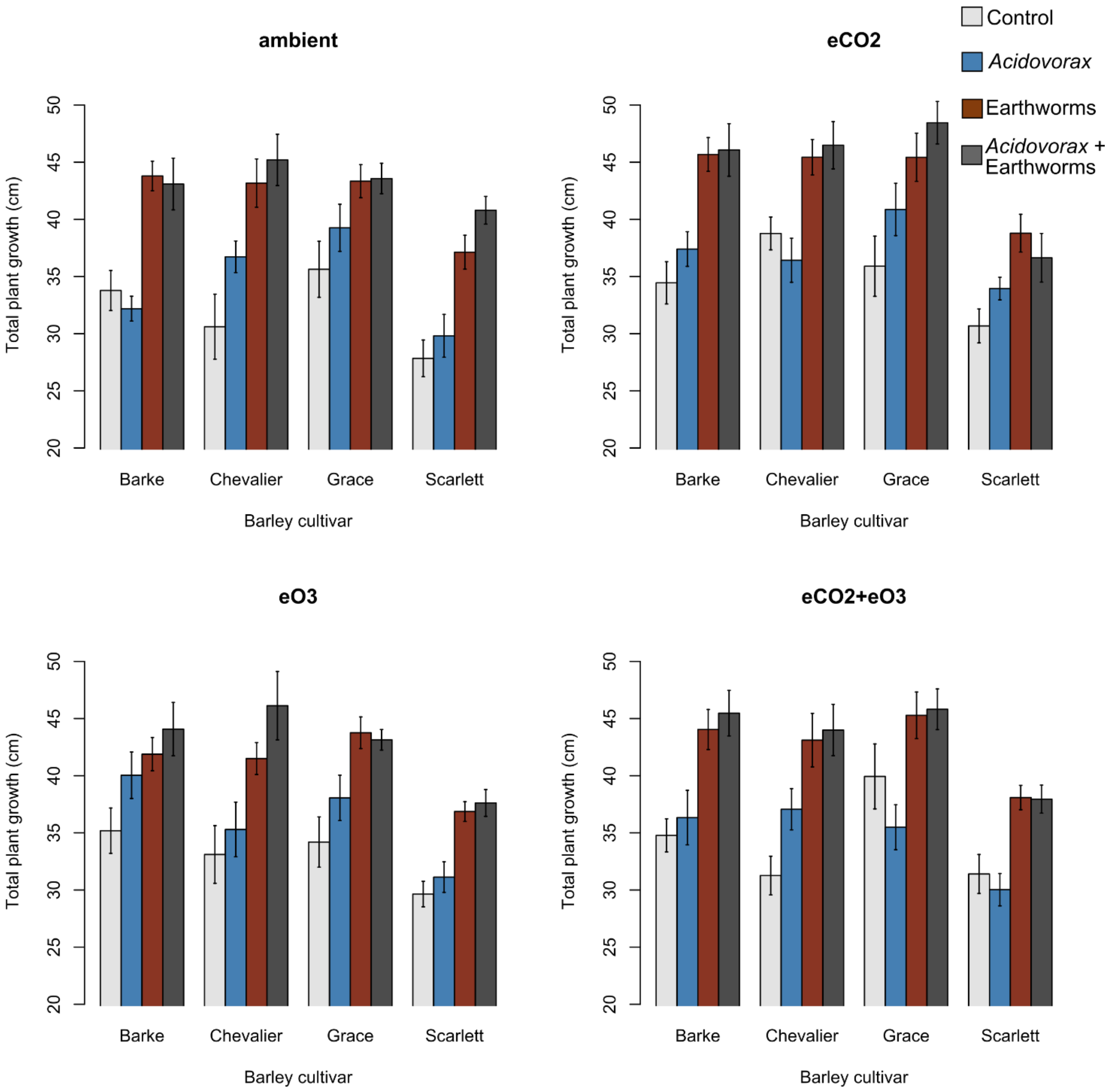

Figure S5. Total plant growth (shoot + root growth) for each barley cultivar within each abiotic environment, across the different *Acidovorax* and earthworm treatments. Error bars +/- 1SE.

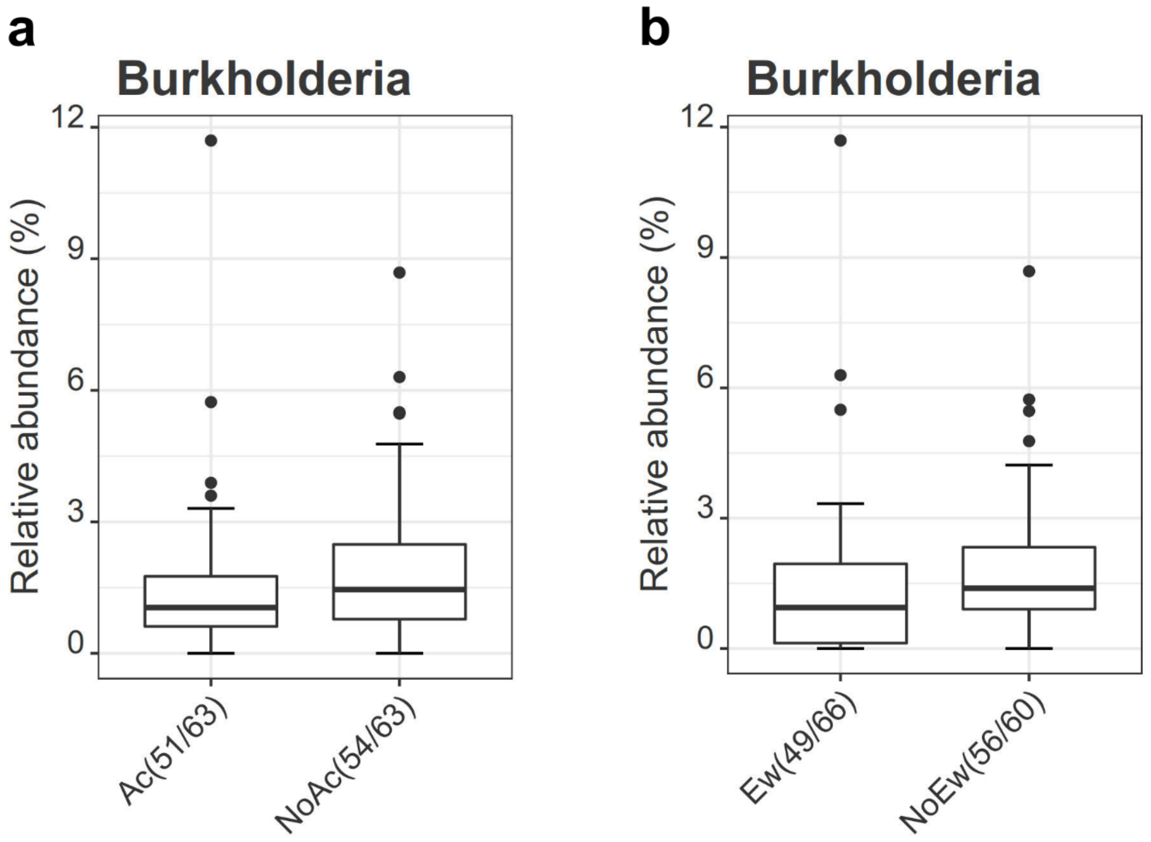

Figure S6. Serial Group-Comparison depicted as box plots for *Burkholderia* abundance, which was found to be significantly lower for treatments (a) with *A. radicis* (Ac, p=0.037) and (b) with earthworms (Ew, p=0.012) compared to samples without the respective organisms (NoAc, NoEw). The bottom and top of the box indicate the first and third quartiles, the line inside the box the median and the ends of the whiskers the 10th and 90th percentile values. Outliers are plotted as circles. The second number in brackets indicates how many samples contained sequences allocated to the genus, the first is the number of samples with the respective treatment (i.e. Acidovorax/no Acidovorax, earthworm/no earthworms) adding up to a total of 126 samples. The 4 control samples were not included in this analysis.

Table S1. Summary of linear model results for the plant and aphid response variables

		Seedling viability (d5-8)			Seedling viability without cv Chevallier			Plant growth (d8-d22)			Root growth (d5-d22)			Aphid load (per cm of plant)		
	**df**	**F**	**P**		**F**	**P**		**F**	**P**		**F**	**P**		**F**	**P**	
as.factor(Run)	2	**105.15**	**<0.001**	***	**112.83**	**<0.001**	***	**38.46**	**<0.001**	***	**27.51**	**<0.001**	***	**24.01**	**<0.001**	***
plant growth variable	1	0.06	0.810		0.03	0.854		**8.25**	**0.004**	**	**51.19**	**<0.001**	***	na	na	
Cultivar	3	**90.65**	**<0.001**	***	**112.27**	**<0.001**	***	**55.31**	**<0.001**	***	**11.03**	**0.001**	***	**2.47**	**0.061**	.
Acidovorax	1	1.98	0.160		11.17	**<0.001**	***	0.41	0.521		**18.29**	**0.001**	***	0.46	0.500	
Earthworms	1	na	na		-	-		**968.66**	**<0.001**	***	**9.98**	**0.002**	**	**104.29**	**<0.001**	***
Aphids	1	na	na		-	-		**58.27**	**<0.001**	***	**3.73**	**0.054**	.	na	na	
eCO2	1	0.78	0.376		0.01	0.967		**18.35**	**<0.001**	***	0.03	0.861		**5.80**	**0.016**	**
eO3	1	**12.86**	**<0.001**	***	**6.35**	**0.012**	*	**5.01**	**0.025**	*	0.01	0.937		0.90	0.343	
Cultivar:Acidovorax	3	**4.83**	**0.002**	**	0.06	0.945		0.50	0.682		0.45	0.719		-	-	
Cultivar:Aphids	3	na	na		na	na		**5.44**	**0.001**	**	0.57	0.638		na	na	
Cultivar:Earthworms	3	na	na		na	na		**2.70**	**0.045**	*	0.67	0.568		-	-	
Cultivar:eCO2	3	1.53	0.205		0.86	0.423		0.99	0.396		1.10	0.349		**2.54**	**0.036**	*
Cultivar:eO3	3	1.10	0.350		0.73	0.480		0.84	0.474		1.26	0.288		-	-	
Acidovorax:Aphids	1	na	na		na	na		0.46	0.498		0.21	0.650		na	na	
Acidovorax:Earthworms	1	na	na		na	na		-	-		0.48	0.489		-	-	
Acidovorax:eCO2	1	**-**	**-**		**4.71**	**0.030**	*	0.41	0.522		2.65	0.104		**4.42**	**0.036**	*
Acidovorax:eO3	1	0.09	0.770		0.01	0.926		**3.21**	**0.074**	.	0.57	0.452		-	-	
Aphids:eCO2	1	na	na		na	na		0.18	0.669		0.18	0.668		na	na	
Aphids:eO3	1	na	na		na	na		0.39	0.531		0.02	0.902		na	na	
Earthworms:Aphids	1	na	na		na	na		0.32	0.569		0.32	0.572		-	-	
Earthworms:eCO2	1	na	na		na	na		2.05	0.153		0.02	0.900		**8.31**	**0.004**	**
Earthworms:eO3	1	na	na		na	na		2.30	0.130		0.01	0.925		-	-	
eCO2:eO3	1	1.34	0.247		2.29	0.131		**2.88**	**0.090**	.	0.92	0.338		-	-	
Cultivar:Acidovorax:Earthworms	3	na	na		na	na		**-**	**-**		0.44	0.726		-	-	
Cultivar:Acidovorax:eCO2	3	**-**	**-**		**-**	**-**		0.64	0.586		0.12	0.951		-	-	
Cultivar:Acidovorax:eO3	3	**3.37**	**0.018**	*	**5.28**	**0.005**	**	1.57	0.195		0.91	0.433		-	-	
Cultivar:Aphids:eCO2	3	na	na		na	na		-	-		-	-		na	na	

Cultivar:Aphids:eO3	3	na	na		na	na		**3.96**	**0.008**	**	-	-		na	na
Cultivar:Earthworms:Aphids	3	na	na		na	na		**5.16**	**0.002**	**	**2.64**	**0.048**	*	-	-
Cultivar:Earthworms:eCO2	3	na	na		na	na		**2.47**	**0.061**	.	0.50	0.680		-	-
Cultivar:Earthworms:eO3	3	na	na		na	na		-	-		0.06	0.983		-	-
Cultivar:eCO2:eO3	3	**3.34**	**0.019**	*	**4.65**	**0.010**	**	**2.16**	**0.091**	.	0.01	0.999		-	-
Acidovorax:Aphids:eCO2	1	na	na		na	na		0.07	0.787		0.96	0.329		na	na
Acidovorax:Aphids:eO3	1	na	na		na	na		0.03	0.870		2.15	0.143		na	na
Acidovorax:Earthworms:Aphids	1	na	na		na	na		-	-		0.05	0.822		na	na
Acidovorax:Earthworms:eCO2	1	na	na		na	na		-	-		0.08	0.771		-	-
Acidovorax:Earthworms:eO3	1	na	na		na	na		-	-		0.00	0.995		-	-
Acidovorax:eCO2:eO3	1	-	-		-	-		0.47	0.492		1.42	0.233		-	-
Aphids:eCO2:eO3	1	na	na		na	na		0.25	0.616		-	-		na	na
Earthworms:Aphids:eCO2	1	na	na		na	na		-	-		1.31	0.253		na	na
Earthworms:Aphids:eO3	1	na	na		na	na		**4.59**	**0.032**	*	2.30	0.130		na	na
Earthworms:eCO2:eO3	1	na	na		na	na		-	-		**3.91**	**0.048**	*	-	-
Cultivar:Acidovorax:Earthworms:eCO2	3	na	na		na	na		-	-		0.35	0.786		-	-
Cultivar:Acidovorax:Earthworms:eO3	3	na	na		na	na		-	-		0.35	0.792		-	-
Cultivar:Acidovorax:eCO2:eO3	3	-	-		-	-		**2.17**	**0.090**	.	0.95	0.416		-	-
Cultivar:Earthworms:Aphids:eCO2	3	na	na		na	na		-	-		-	-		na	na
Cultivar:Earthworms:eCO2:eO3	3	na	na		na	na		-	-		0.40	0.756		-	-
Acidovorax:Aphids:eCO2:eO3	1	na	na		na	na		**4.02**	**0.045**	*	-	-		na	na
Acidovorax:Earthworms:Aphids:eCO2	1	na	na		na	na		-	-		2.03	0.154		na	na
Acidovorax:Earthworms:Aphids:eO3	1	na	na		na	na		-	-		-	-		na	na
Acidovorax:Earthworms:eCO2:eO3	1	na	na		na	na		-	-		0.20	0.651		-	-
Cultivar:Acidovorax:Earthworms:eCO2:eO3	3	na	na		na	na		-	-		**4.05**	**0.007**	**	-	-
Residual degrees of freedom		Res df=959			Res df=685			Res df=923			Res df=902			Res df=434	
Weighting factor used to correct for heteroscedasticity		weighting: varfunc(Cultivar)			weighting: varfunc(Cultivar)			weighting: varfunc(Cultivar)			weighting: varfunc(Cultivar)			weighting: varfunc(Cultivar*Acido*Ew)	

Values in bold show significant P<0.10, values in grey are kept in the minimal adequate model but not-significant at P>0.10, '-' means that the term did not remain in the minimal adequate model and thus was removed, 'na' means the term was not included in the model (e.g. no aphid or earthworms terms for seedling viability as they were not introduced at that stage).

*** P<0.001, ** P<0.01, * P<0.05, • P<0.10

Table S2. Summary of matched pairs analysis for effect of *Acidovorax* on plant and aphid response variables

Paired data: *Acidovorax* effect		Seedling viability (d5-8)			Plant growth (d8-d22)			Root growth (d5-d22)			Aphid density		
	Df	F	P		F	P		F	P		F	P	
as.factor(Run)	2	-	-		-	-		**6.13**	**0.002**	**	**5.72**	**0.004**	**
Cultivar	3	**8.32**	**<0.001**	***	-	-		0.72	0.543		-	-	
Earthworms	1	na	na		1.23	0.267		0.54	0.462		0.95	0.330	
Aphids	1	na	na		0.33	0.564		0.16	0.691		na	na	
eCO2	1	3.45	0.064	.	0.45	0.501		2.63	0.105		**3.98**	**0.047**	*
eO3	1	0.07	0.794		**4.43**	**0.036**	*	1.10	0.295		1.05	0.306	
Cultivar:Earthworms	3	na	na		-	-		0.77	0.513		-	-	
Cultivar:eCO2	3	-	-		-	-		0.32	0.810		-	-	
Cultivar:eO3	3	**4.08**	**0.007**	**	-	-		1.81	0.145		-	-	
Aphids:eCO2	1	na	na		-	-		**3.11**	**0.078**	.	na	na	
Aphids:eO3	1	na	na		-	-		**2.74**	**0.099**	.	na	na	
Earthworms:Aphids	1	na	na		2.29	0.131		-	-		na	na	
Earthworms:eCO2	1	na	na		0.23	0.628		0.29	0.592		-	-	
Earthworms:eO3	1	na	na		1.38	0.240		0.02	0.881		**3.00**	**0.085**	.
eCO2:eO3	1	-	-		0.02	0.894		**4.03**	**0.045**	*	-	-	
Cultivar:Earthworms:eCO2	3	na	na		-	-		0.38	0.766		-	-	
Cultivar:Earthworms:eO3	3	na	na		-	-		0.21	0.893		-	-	
Cultivar:eCO2:eO3	3	-	-		-	-		1.06	0.366		-	-	
Earthworms:eCO2:eO3	1	na	na		**5.39**	**0.021**	*	0.90	0.344		-	-	
Cultivar:Earthworms:eCO2:eO3	3	na	na		-	-		**3.90**	**0.009**	**	-	-	
**Residual degrees of freedom**		Res df=465			Res df=464			Res df=437			Res df=209		
**Weighting factor used to correct for** heteroscedasticity		weighting: varfunc(Cultivar)			weighting: varfunc(Cultivar)			weighting = none			weighting = none		

Values in bold show significant P<0.10, values in grey are kept in the minimal adequate model but not-significant (P>0.10), '-' means that the term did not remain in the minimal adequate model and thus was removed, 'na' means the term was not included in the model (e.g. no aphid or earthworms terms for seedling viability as they were not introduced at that stage).

*** P<0.001, ** P<0.01, * P<0.05, • P<0.10
